# Cerebellar-dependent associative learning is impaired in very preterm born children and young adults

**DOI:** 10.1038/s41598-017-18316-8

**Published:** 2017-12-21

**Authors:** Liliane Tran, Britta M. Huening, Olaf Kaiser, Bernd Schweiger, Selma Sirin, Harald H. Quick, Ursula Felderhoff-Mueser, Dagmar Timmann

**Affiliations:** 1 Department of Paediatrics I, Neonatology, Paediatric Intensive Care, Paediatric Neurology, University Hospital Essen, University of Duisburg-Essen, Essen, Germany; 2 Erwin L. Hahn Institute for Magnetic Resonance Imaging, University of Duisburg-Essen, Essen, Germany; 3 Institute of Diagnostic and Interventional Radiology and Neuroradiology, University Hospital Essen, University of Duisburg-Essen, Essen, Germany; 4 High Field and Hybrid MR Imaging, University Hospital Essen, University of Duisburg-Essen, Essen, Germany; 5 Department of Neurology, University Hospital Essen, University of Duisburg-Essen, Essen, Germany

## Abstract

Preterm birth incorporates an increased risk for cerebellar developmental disorders likely contributing to motor and cognitive abnormalities. Experimental evidence of cerebellar dysfunction in preterm subjects, however, is sparse. In this study, classical eyeblink conditioning was used as a marker of cerebellar dysfunction. Standard delay conditioning was investigated in 20 adults and 32 preschool children born very preterm. Focal lesions were excluded based on structural magnetic resonance imaging. For comparison, an equal number of matched term born healthy peers were tested. Subgroups of children (12 preterm, 12 controls) were retested. Preterm subjects acquired significantly less conditioned responses (CR) compared to controls with slower learning rates. A likely explanation for these findings is that preterm birth impedes function of the cerebellum even in the absence of focal cerebellar lesions. The present findings are consistent with the assumption that prematurity results in long-term detrimental effects on the integrity of the cerebellum. It cannot be excluded, however, that extra-cerebellar pathology contributed to the present findings.

## Introduction

Eleven per cent of all live births worldwide occur before term (<37 weeks of gestation), 2.5% before 32 weeks of gestation, with an observable upward trend^1^. Substantial progress in neonatal care has decreased mortality in this vulnerable patient population over the last decades^2^. To date, not only the large majority of very immature infants survive, but also the incidence of classic cystic periventricular leukomalacia and severe supratentorial haemorrhage associated with cerebral palsy has significantly decreased^3^. In recent years, long-term cognitive and socio-emotional sequelae have become increasingly evident in prematurely born children^4–6^.

Neurodevelopmental disabilities in former preterm infants are most commonly attributed to subtle diffuse white matter injury in the cerebrum^7^. More recent findings suggest that cerebellar pathology likely plays an important role. Cerebellar haemorrhage is frequent in very preterm children and occurs in up to 19%^8,9^. The most prevalent cerebellar pathology, however, is cerebellar growth failure. It is observed in 50% of the very immature population and is independent of cerebellar lesions^10,11^. The cerebellum grows rapidly during the period from 28 weeks’ postconceptional age to term^12^, with a 5-fold increase in volume and a 30-fold increase of surface area^13,14^. It even exceeds the growth rate of the cerebral hemispheres during the last trimester of pregnancy^12^. This rapid growth rate makes the cerebellum vulnerable to focal injuries and developmental abnormalities during the last trimester of pregnancy.

In a seminal study, Limperopoulos and colleagues observed neurologic abnormalities in 66% of 35 preterm infants at a mean age of 32 months following isolated cerebellar haemorrhage. Abnormalities included motor impairment (48%), cognitive deficits (40%), learning and behavioural problems (34%) and indications of autism (37%)^15^. These findings agree with other studies that found not only motor, but also cognitive, emotional and behavioural abnormalities in cerebellar disease, in particular when it is acquired at a young age^16,17^. Likewise, reduction in cerebellar volume in preterm children without structural injury was positively correlated with cognitive outcome from early childhood through adolescence^18,19^. In particular, smaller hemispheres were associated with reduced executive, visuospatial and language functions and motor skills^20^. At school age cerebellar injury and growth failure were associated with attention and learning problems^21,22^.

To date, however, few studies provided unequivocal evidence for cerebellar dysfunction on a behavioural level in very preterm children lacking focal disease^23^. In addition, in these children motor deficits are usually subtle, and standard motor tests do not allow differentiating between cerebellar and cerebral involvement^23–25^. Likewise, although certain patterns of cognitive dysfunction have been associated with cerebellar disease^17,26^, standard neuropsychological testing cannot differentiate between cerebellar and, e.g. prefrontal contributions. Furthermore, as of yet, most studies included preterm children in early infancy or childhood and it is unclear whether cerebellar dysfunction remains until late adolescence and adulthood.

In the present study, classical eyeblink conditioning was used as a marker of cerebellar dysfunction. Eyeblink conditioning is a form of associative learning, which depends on the integrity of the cerebellum. In this paradigm, an aversive stimulus directed to the eye (e.g. an air puff) serves as the unconditioned stimulus (US)^27^. The US results in eyelid closure as the unconditioned response (UR). When a US is repeatedly preceded by an initially neutral conditioned stimulus (CS), e.g. a tone, healthy subjects learn to close their eyes in response to the tone and prior onset of the air puff, that is, they learn to elicit a conditioned response (CR). The neural substrates of classical eyeblink conditioning have been studied in great detail^28–31^. The cerebellar cortex, in particular the intermediate part of lobule VI, and the cerebellar nuclei, more specifically the interposed nuclei, are known to be critically involved^27,29,32^. Eyeblink conditioning is severely reduced or even absent in adults with focal or degenerative cerebellar disease^33–35^. More importantly, eyeblink conditioning has been shown to be a sensitive tool to detect cerebellar dysfunction in disorders in which cerebellar motor signs are absent or subtle. This includes patients with essential tremor^36^, migraine^37^, attention deficit hyperactivity disorder^38^, and dyslexia^39,40^.

In the present study classical delay eyeblink conditioning was investigated in two cohorts of former preterm infants born before 32 weeks gestation at preschool age and young adults. Participants with focal cerebellar and cerebral lesions were excluded based on structural brain magnetic resonance imaging (MRI). Impaired eyeblink conditioning would strengthen the hypothesis of cerebellar developmental alterations following preterm birth. Furthermore, it was investigated whether potential cerebellar dysfunction detected at early school age prevails into young adulthood. It was hypothesized that functional integrity of the cerebellum is persistently impaired in subjects born preterm.

## Results

### Preterm born adults

#### Clinical ataxia scores

Neurological examination showed no (n = 15/20) or mild abnormalities (n = 5/20) in preterm subjects and none in controls. Abnormalities were not specific for cerebellar ataxia. Total Scale for the Assessment and Rating of Ataxia (SARA) and International Cooperative Ataxia Rating Scale (ICARS) ataxia scores were low and ranged between 1 and 2 (maximum SARA score = 40, maximum ICARS score = 100)^41,42^, (Table 1).

**Table 1:** Clinical characteristics in preterm born adults and matched controls.

	Prenatal adults (n = 20)	Control adults (n = 20)
**Demographic characteristics**		
Age - mean (range)	19.4 (18.1–24.5)	19.9 (18.5–23.5)
Sex - male/female - n	10/10	10/10
Handedness - right/left/both - n	19/1/0	20/0/0
**Perinatal characteristics**		
Gestational age at birth in weeks - mean (range)	28.9 (23.3–32.0)	39.4 (37.0–41.0)
Birth weight in grams - mean (range)	1219 (650–1970)	3471 (2540–4300)
Small for gestational age (<10th centile) - n	3	2
5-min APGAR score - median (range)	7 (4–9)	10 (9–10)
10-min APGAR score - median (range)	8 (7–10)	10 (9–10)
Umbilical artery pH - median (range)	7.25 (7.02–7.34)	7.31 (7.15–7.43)
AIS – n	4	0
Antenatal steroids - no/yes/unknown - n	12/4/4	20/0/0
**Postnatal characteristics**		
Proven sepsis - no/yes/unknown - n	9/9/2	19/0/1
Postnatal steroids - no/yes/unknown - n	13/4/3	20/0/0
Bronchopulmonary dysplasia – no/yes/ unknown – n	12/6/2	n.a.
Necrotizing enterocolitis – no/yes/unknown - n	18/0/2	n.a.
Patent ductus arteriosus – no/yes/unknown - n	10/8/2	n.a.
Retinopathy of prematurity – no/yes/unknown - n	10/9/1	n.a.
Any conducted operation – no/yes/unknown - n	15/4/1	16/1/3
Cerebral MRI at study date*		
Intraventricular haemorrhage - n	0	0
Grade I – n	0	0
Grade II – n	0	0
Ventricular dilatation** - n	12	5
Yes, mild – n	9	3
Yes, moderate – n	3	2
Punctate cerebral lesions - n	0	0
White matter injury - n	0	0
Volumetric analysis of MRI data***		
Total intracranial volume (TICV) (mm^3^) – mean (range)	1486.8 (1238.0–1750.0)	1515.9 (1182.0–1846.0)
Cerebral volume (mm^3^) – mean (range)	1223.8 (1008.0–1467.2)	1255.2 (963.9–1538.2)
Cerebellar volume (mm^3^) – mean (range)	149.7 (126.6–174.8)	154.9 (131.7–189.0)
Cerebral volume/% TICV – mean (range)	83.1 (78.1–87.2)	83.0 (78.5–89.3)
Cerebellar volum/% TICV – mean (range)	10.1 (9.0–11.5)	10.3 (8.9–12.1)
**Cerebral ultrasonography (postnatal period)**		
Intraventricular haemorrhage - n	5	n.a.
Grade I - n	2	n.a.
Grade II - n	3	n.a.
Periventricular leukomalacia at term-equivalent age - n	0	n.a.
**Ataxia scores at study date**		
ICARS score - mean (range)	0.3 (0.5–2)	0
SARA score - mean (range)	0.27 (1–2)	0
Education****		
Level I	2	0
Level II	9	7
Level III	9	13

AIS = amnion infection syndrome; APGAR = method to score the postnatal adaptation of a newborn^107^; ICARS = International Cooperative Ataxia Rating Scale (minimum score = 0; maximum score = 100)^41^; n = number; n.a. = not applicable; SARA = Scale for the Assessment and Rating of Ataxia (minimum score = 0, maximum score = 40)^42^; *Two participants of the control group refused to have a brain MRI; **Note, that findings are based on visual inspection. Based on visual inspection, a similar proportion of ventricular dilatation has been reported in young and healthy subjects in the literature^108^; ***Volumetric analysis of MRI data was performed semi-automatically in T1 weighted MP2RAGE images (3 Tesla) using ECCET Software (http://www.eccet.de) as described previously^109^. Group differences were not significant (all p values > 0.28; unpaired t-tests); ****The German school system has three levels of examination; level III qualifies for university entrance.

#### CR incidence

**Figure 1:**
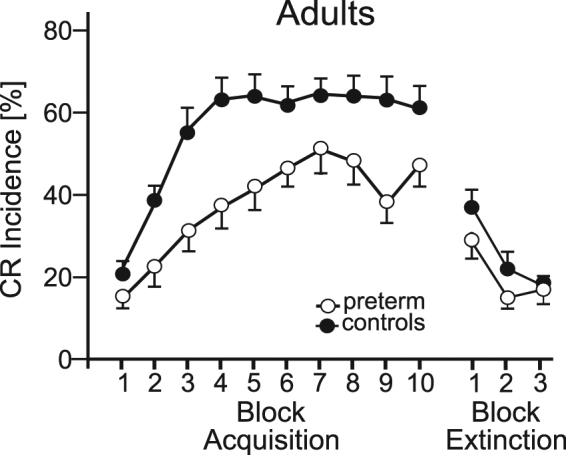
Acquisition and extinction of conditioned eyeblinks in adults. Mean percentage conditioned response (CR) incidence and standard error (SE) are shown in preterm born adults (ο) and control adults (•). The first ten blocks are acquisition blocks, the last three blocks extinction blocks. Each block corresponds to ten trials. Both groups significantly increased CR incidence across blocks. The group and block by group interaction effects were significant. There was a significant effect of extinction, but no difference between groups (see Supplementary Table 1 for details).

Control subjects increased the number of conditioned responses during the first four acquisition blocks with no additional increase during the last six acquisition blocks (• in Fig. 1). Preterm born adults also increased the number of conditioned responses. The increase across the first four blocks, however, was less compared to controls. The increase continued up to the seventh block (ο in Fig. 1). Preterm born adults were not only slower in CR acquisition compared to controls, they also acquired less conditioned responses. Group mean total CR incidences were 38.5% (SD 18.8%) in preterm born adults and 57.5% (SD 18%) in matched controls. ANOVA with repeated measures showed a significant block effect [F (9, 342) = 30.18, *P* < 0.001], that is both groups increased CR incidence across blocks. Group effect and block by group interaction were significant [group effect: F (1, 38) = 10.74, *P* = 0.002, block by group interaction: F (9, 342) = 2.19, *P* = 0.02]. These findings are further illustrated in Supplementary Fig. 1a showing EMG recordings of the 100 paired CS-US acquisition trials in a characteristic preterm born adult and age-matched control subject. The preterm subject (male, age 20 yrs) needed 26 trials to show the first conditioned response, whereas the first CR was present in trial 3 in the control subject (female, age 23 yrs). In addition, the preterm born adult acquired less CRs than the control (total CR incidence preterm subject: 40%; control subject: 71%).

There was no difference between groups considering extinction. Both control and preterm subjects showed a marked decline in the first extinction block compared to the last acquisition block (Fig. 1). This decline continued across the three extinction blocks. In the last extinction block CR incidence had returned to the value of the first acquisition block. ANOVA with repeated measures showed a significant block effect [F (3, 111) = 48.17, *P* < 0.001]. The group and block by group interaction effects were not significant [group effect F (1, 37) = 2.45, *P* = 0.13, block by group effect, F (3, 111) = 1.47, *P* = 0.23].

#### CR timing and performance

**Figure 2:**
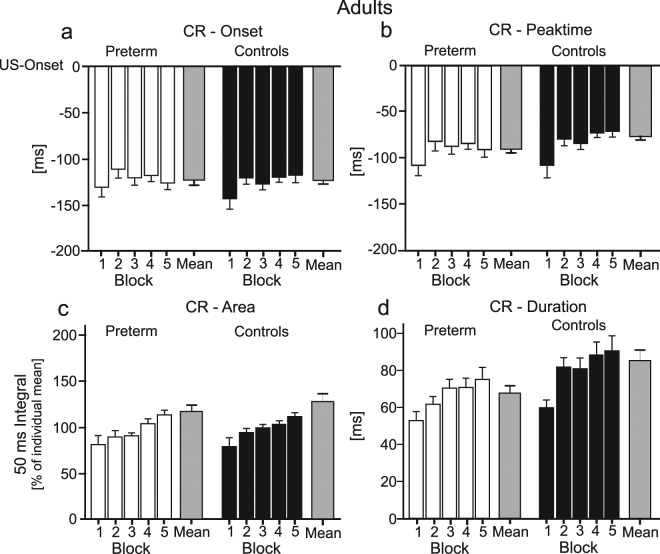
Timing and performance of conditioned eyeblink responses in adults. Means and standard errors (SE) of (**a**) CR onset, (**b**) CR peak time, (**c**) CR area and (**d)** CR duration are shown in preterm born adults (open columns) and control adults (filled columns). Note that (negative) values for CR onset and CR peak time refer to the time prior to the onset of the US (air puff), set as 0 ms. Each block corresponds to 20 CS-US paired acquisition trials. Mean values of all acquired CRs are shown in the grey columns. CR peak time, duration, and area showed a significant change across blocks in both groups (see Supplementary Table 1 for details).

In both preterm and control adults CR onset and CR peak time shifted across acquisition blocks. In the first block of 20 acquisition trials, CR onset and CR peak time occurred earlier, that is further away from the US onset, than in the subsequent four blocks (Fig. 2a,b). In addition, there was an increase of CR duration and CR area (50 ms integral) across blocks in both groups (Fig. 2c,d). ANOVA with repeated measures showed a significant block effect for three of the four parameters [CR peak time: F (4, 136) = 6.14, *P* = 0.001; CR duration: F (4, 136) = 20.68, *P* < 0.001; CR area: F (1, 43) = 10.65, *P* = 0.004], and a block effect on a trend level for CR onset [F (4, 136) = 3.64, *P* = 0.02]. There were no significant group and block by group interaction effects (all *P* values > 0.2; see Table 1 in Supplementary Information for details) except for a group effect in duration on a trend level [F (1, 34) = 4.34, *P* = 0.04]. Mean duration was longer in controls than preterm born adults (Fig. 2d).

#### Alpha responses, unconditioned responses and spontaneous blink rate

The mean total alpha response count was 5.8 (SD 3.99) in the preterm group and 3.7 (SD 1.83) in the control group. The group difference was significant [F (1, 38) = 4.56, *P* = 0.04; ANOVA with repeated measures]. The number of alpha responses did not significantly change across blocks neither in the preterm nor in the control group [block effect: F (9, 342) = 0.65, *P* = 0.69; block by group interaction: F (9, 342) = 1.08, *P* = 0.38]. To exclude that reduced CR acquisition in preterm born adults was caused by ill-timed CRs (i.e., CRs occurring so early that they were identified as alpha responses), analysis of CR acquisition was repeated with alpha responses being considered CRs. Main findings remained the same (see Supplementary Fig. 2).

Mean UR onset, peak time and duration in the unpaired US-only trials did not significantly differ between groups (all *P* values > 0.16; see Table 2 for mean values and Supplementary Table 1 for statistical comparisons).

**Table 2:** UR and CR timing and performance parameters, spontaneous blink rate and alpha response count. Group means ± SD in preterm born adults and children compared to the matched control groups.

	UR onset	UR peak time	UR duration	CR onset	CR peak time	CR duration	CR area (50 ms integral)	Spontaneous blinks (beginning/end)	Alpha responses
	[ms]	[ms]	[ms]	[ms]	[ms]	[ms]	[% individ. mean]	[blinks/min]	[count]
**Adult group (n** = **20)**									
Preterms	57.57 ± 6.73	96.83 ± 13.56	94.46 ± 21.65	−122.09 ± 24.79	−88.92 ± 27.32	68.05 ± 19.17	115.90 ± 30.78	26.7 ± 4.10/26.7 ± 4.9	5.8 ± 3.99
Controls	57.18 ± 7.55	100.66 ± 20.04	90.35 ± 28.37	−122.23 ± 18.48	−76.81 ± 15.83	86.43 ± 22.01	127.19 ± 30.45	29.15 ± 7.01/28.94 ± 9.36	3.7 ± 1.83
**Children group (all, n = 32)**									
Preterms	69.9 ± 8.91	128.50 ± 18.04	115.33 ± 33.86	−154.94 ± 20.43	−130.25 ± 22.75	57.24 ± 12.53	73.77 ± 23.10	14.34 ± 6.4/15.06 ± 7.59	8.28 ± 4.93
Controls	62.69 ± 8.27	113.29 ± 26.63	113.31 ± 27.07	−150.19 ± 22.17	−125.44 ± 23.24	53.89 ± 9.42	73.06 ± 28.99	11.34 ± 7.35/12.69 ± 8.87	6.47 ± 3.97
**Session 1 (n = 12)**									
Preterms	68.34 ± 6.49	124.34 ± 14.54	112.34 ± 25.04	−157.85 ± 19.88	−134.02 ± 21.91	55.49 ± 9.24	96.32 ± 22.11	15.16 ± 5.87/17.83 ± 8.15	5.42 ± 3.37
Controls	60.3 ± 6.91	117.98 ± 22.69	125.02 ± 30.44	−151.45 ± 25.61	−126.38 ± 27.49	53.84 ± 8.53	71.27 ± 18.42	11.41 ± 10.19 /10.58 ± 5.05	5.92 ± 2.64
**Session 2 (n = 12)**									
Preterms	66.16 ± 8.62	112.67 ± 20.75	97.97 ± 27.68	−140.65 ± 28.13	−115.53 ± 31.9	53.59 ± 9.15	99.0 ± 15.35	9.25 ± 5.77/8.91 ± 5.28	4.58 ± 3.8
Controls	63.23 ± 12.94	126.57 ± 28.22	119.50 ± 32.83	−142.85 ± 41.19	−105.38 ± 44.38	72.85 ± 18.51	121.21 ± 18.50	7.16 ± 2.51/7.16 ± 2.79	3.83 ± 2.66

Note that (negative) values for CR onset and CR peak time refer to the time prior to the onset of the US (air puff), set as 0 ms.

Comparison of spontaneous blink rate showed no difference between preterm and control groups, the beginning and end of the session and no significant interaction (all *P* values > 0.18; see Table 2 and Supplementary Table 1).

#### Correlation with birth weight, gestational age and ataxia scores

In the group of preterm born adults total CR incidence showed no significant correlation with birth weight (R = 0.014, *P* = 0.95), gestational age in days (R = 0.007, *P* = 0.98), ICARS score (ρ = −0.3, *P* = 0.2) or SARA score (ρ = −0.25, *P* = 0.29).

### Preterm preschool children

#### Clinical ataxia scores

As expected, mild incoordination and balance problems were present in children at 5–6 years^43,44^ that were more pronounced in preterm children. Total SARA scores ranged from 0–7 in preterm children and 0–3 in controls; total ICARS scores from 0–17 and 0–5, respectively (Table 3). 7/32 preterm children showed total SARA scores which were higher than in the worst performing control child, and 16/32 preterm children total ICARS scores which were higher than in the worst performing control child. No participant showed any other abnormal neurological signs.

**Table 3:** Clinical characteristics in preterm children and matched controls.

	Preterm children (n = 32)	Control children (n = 32)
**Demographic characteristics**		
Age − mean (range)	5.8 (5.0–6.6)	5.8 (5.0–6.7)
Sex - male/female - n	16/16	16/16
Handedness - right/left/both - n	22/9/1	31/1/0
**Perinatal characteristics**		
Gestational age at birth in weeks - mean (range)	28.0 (23.7–31.7)	39.4 (37.0–41.6)
Birth weight in grams - mean (range)	1047 (540–1670)	3397 (2630–4060)
Small for gestational age (<10th centile) - n	2	1
5-min APGAR score - median (range)	8 (5–9)	10 (8–10)
10-min APGAR score - median (range)	9 (6–10)	10 (9–10)
Umbilical artery pH - median (range)	7.33 (7.13–7.45)	7.27 (7.0–7.48)
AIS - n	12	0
Antenatal steroids - no/yes/unknown - n	4/28/0	32/0/0
**Postnatal characteristics**		
Proven sepsis - no/yes/unknown - n	18/14/0	32/0/0
Postnatal steroids - no/yes/unknown - n	5/27/0	32/0/0
Bronchopulmonary dysplasia - no/yes/unknown - n	13/9/0	32/0/0
Necrotizing Enterocolitis - no/yes/unknown - n	28/0/3	32/0/0
Patent ductus arteriosus – no/yes/unknown - n	17/15/0	32/0/0
Retinopathy of prematurity – no/yes/unknown - n	15/17/0	32/0/0
Any conducted operation - no/yes/unknown - n	18/14/0	32/0/0
**Cerebral MRI (at term)**		
Intraventricular haemorrhage - n	5	n.a.
Grade I – n	1	n.a.
Grade II - n	4	n.a.
Ventricular dilatation - n	14	n.a.
Yes, mild - n	12	n.a.
Yes, moderate - n	2	n.a.
Punctate cerebral lesions - n	3	n.a.
White matter injury - n	2	n.a.
Mild white matter injury - n	2	n.a.
Moderate white matter injury - n	0	n.a.
Simple MRI brain metrics*		n.a.
Cerebral biparietal width (mm) – mean (range)	75.3 (67.2–90.3)	n.a.
<1^st^ centile (fetal MRI)** - n	31	n.a.
out of interquart. range (neonatal MRI)*** - n	1	n.a.
Transcerebellar diameter (mm) – mean (range)	51.7 (45.3–59.9)	n.a.
<1^st^ centile (fetal MRI)** - n	11	n.a.
out of interquart. range (neonatal MRI)*** - n	2	n.a.
**Ataxia scores at study date**		
ICARS score - mean (range)	6 (0–17)	1 (0–5)
SARA score - mean (range)	2.47 (0–7)	0.28 (0–3)
**Education**		
Kindergarten – n	28	22
Primary school - n	4	10

AIS = amnion infection syndrome; APGAR = method to score the postnatal adaptation of a newborn^107^; * Simple brain metrics were applied according to Nguyen The Tich *et al*.^110^; ** Measurements were compared to reference values of fetal MRI^111^, *** and to MRI values in preterm infants at term equivalent age reported by Brouwer *et al*.^112^; ICARS = International Cooperative Ataxia Rating Scale (minimum score = 0; maximum score = 100)^41^; n = number; n.a. = not applicable; SARA = Scale for the Assessment and Rating of Ataxia (minimum score = 0, maximum score = 40)^42^.

#### CR incidence

**Figure 3:**
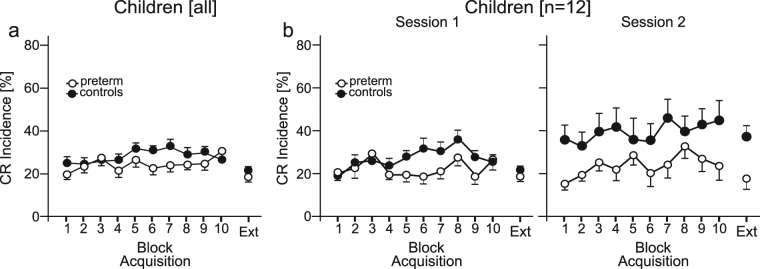
Acquisition and extinction of conditioned eyeblinks in children. Mean percentage conditioned response (CR) incidence and standard error (SE) are shown in preterm children (ο) and control children (•). Data is shown in (**a**) the groups of all children (n = 32 per group) and (**b**) in the subgroups tested twice (session 1 and session 2; n = 12 per group). The first ten blocks are acquisition blocks. Ext = extinction block. Each block corresponds to ten trials. Considering the group of all children, there was no significant block, group or interaction effect during acquisition. Extinction effect was significant in both groups. Considering the subgroups of 12 preterm children, block and group effects were significant during acquisition. Both groups showed significant effects of extinction with no difference between groups (see Supplementary Table 2 for details).

Considering the group of all 32 preterm preschool children and controls, learning effects were low (Fig. 3a). Increase of CR incidence across blocks was small, with CR incidences being higher in the second half of the acquisition phase in controls compared to preterm children. Mean total CR incidence was low in both groups [preterm children: 23.8% (SD 9.2), controls: 26.9% (SD 7.6)]. Both groups showed a decrease of CR incidences in the extinction block compared to the last acquisition block. In the acquisition phase, the block effect did not become significant, that is there was no significant increase of CR incidences across the ten acquisition blocks [block effect: F (9, 558) = 1.50, *P* = 0.14]. Likewise, the group effect did not become significant [F (1, 62) = 2.19, *P* = 0.14] and there was no significant block by group interaction effect [F (9, 558) = 0.96, *P* = 0.47]. ANOVA with repeated measures showed a significant difference comparing the last acquisition and the extinction block [F (1, 62) = 14.17, *P* < 0.001]. The significant decline during extinction indicates that some learning has occurred during acquisition. Group and group interaction effects were not significant [group effect: F (1, 62) = 0.003, *P* = 0.95; block by group interaction: F (1, 62) = 1.57, *P* = 0.21].

In the subgroups of twelve preterm children and matched controls tested twice, learning effects in both groups and differences between groups became more obvious (Fig. 3b). In the first testing session, same as for the group of all 32 subjects, both groups showed a small increase of CR incidence across blocks, which was more prominent in controls (•) than preterm children (ο). Controls showed a decline of CR incidences at the end of the first session. This finding has also been observed in healthy adults, and may be due to decreased alertness at the end of an one hour session^35,45^. In the second session, controls but not preterm children showed CR incidences in the first acquisition block which were significantly higher compared to the last acquisition block of session 1 [session by group interaction: F(1,22) = 9.78, p = 0.005] and within the range of the last acquisition blocks of the first session. In the second session, both groups showed a slight increase of CR incidences across blocks. Total CR incidences were smaller in preterm children compared to matched controls in the second session [first session: preterm: 23.8% (SD 9.2), controls: 26.9% (SD 7.6); second session: preterm: 23.5% (SD 12.2)], controls: 44.9% (SD 24.5)]. Considering acquisition, ANOVA with repeated measures showed a significant block effect [F (9, 198) = 3.20, *P* = 0.001]. The group effect was significant [F (1, 22) = 6.57, *P* = 0.02]. The session effect (session 1 vs. 2) was close to significance [F (1, 22) = 4.07, *P* = 0.06]. There were no significant block by group, session by group, session by block and session by block by group interaction effects (all *P* values > 0.11, statistical data are summarized in Supplementary Table 2). Thus both groups acquired conditioned responses, but total CR incidence was significantly less in preterm children compared to controls.

These findings are further illustrated in Supplementary Fig. 1b showing EMG recordings of the 100-paired CS-US acquisition trials in a characteristic preterm child (male, age 6 yrs) and control child (female, age 5 yrs). In session 1, both children did acquire few CRs (total CR incidence: control child: 22%, preterm child: 24%). In the second session, both subjects acquired more CRs than in the first. Total CR incidence was higher in the control child (53%) compared to the preterm child (27%).

Both subgroups showed significant effects of extinction [last block of acquisition vs. extinction block: F (1, 22) = 11.31, *P* = 0.003]. The block by group interaction effect was not significant [F (1, 22) = 0.01, *P* = 0.94], thus extinction did not differ between groups. Session by group effect [F (1, 22) = 4.83, *P* = 0.04] was significant. The group and session effects were close to significance [group: F (1, 22) = 3.92, *P* = 0.06; session: F (1, 22) = 4.01, *P* = 0.06]. The latter differences reflect the higher CR incidence in the last acquisition block in session 2 in control children.

#### CR timing and performance

**Figure 4:**
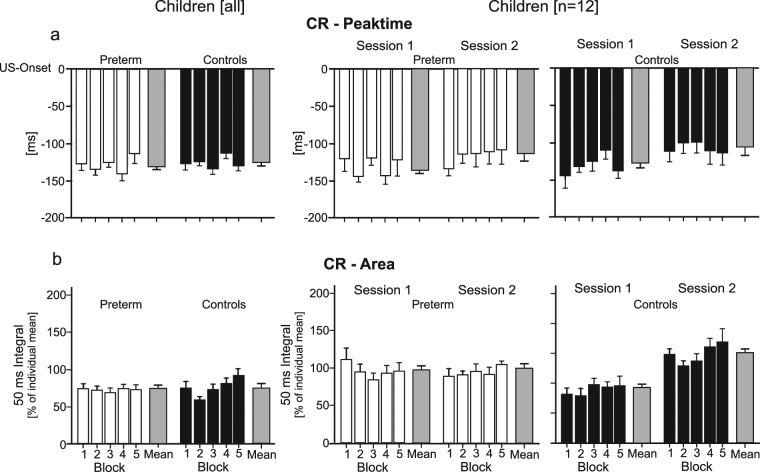
Timing and performance of conditioned eyeblink responses in children. Means and standard errors (SE) of (**a**) CR peak time and (**b**) CR area are shown in preterm children (open columns) and control children (filled columns). Data is shown in the groups of all children (first column; n = 32 per group) and in the subgroups tested twice (second column: session 1, third column: session 2; n = 12 per group). Note that (negative) values for CR peak time refer to the time prior to the onset of the US (air puff), set as 0 ms. Each block corresponds to 20 CS-US paired acquisition trials. Mean values of all acquired CRs are shown in the grey column. In the subgroups tested twice, there was a significant session effect considering CR peaktime, and a significant session by group interaction and session effect considering CR area (see Supplementary Table 2 for details).

Considering the group of all preterm children and controls tested in one session, CR onset and CR peak time showed no obvious change across acquisition blocks (Fig. 4a, see also Supplementary Fig. 3a). In both preterm children and controls, mean CR onset and peak time occurred earlier, that is further away from US onset than in adult controls (cf. Supplementary Fig. 1 and Table 2). In the subgroup of children tested twice, CR onset and peak time tended to occur later in the second session that is closer to the US, in both preterm children and controls. The difference was most obvious for CR peak time and in control children. Analysis of variance showed a significant session effect [F (1, 20) = 7.68, *P* = 0.01], and a session by block by group interaction effect on a trend level considering CR peak time [F (4, 80) = 2.75, *P* = 0.05], but no significant effects considering CR onset. All other effects were not significant (all *P* values > 0.08).

Control children, but not preterm children, increased CR area across acquisition blocks. The increase across acquisition blocks was small in the group of all 32 control children tested in one session (Fig. 4b). The increase in controls and the lack of increase in preterm children was most obvious comparing the first and second session in the subgroups tested twice. Considering all children tested in one session, there were no significant group, block or block by group interaction effects (all *P* values > 0.19; Supplementary Table 2). In the subgroup of children tested twice, the session by group interaction [F (1, 18) = 12.61, *P* = 0.002] was significant showing that controls but not preterm children increased CR area in the second session compared to the first. The session effect was significant [F (1, 18) = 14.10, *P* = 0.001] driven by the prominent increase in controls. Group, block, block by group, session by block, session by block by group (interaction) effects were not significant (all *P* values > 0.52). Likewise, control children, but not preterm born children, increased CR duration across acquisition blocks (see Supplementary Fig. 3b and Supplementary Table 2).

#### Alpha responses, unconditioned responses and spontaneous blink rate

The mean total alpha response count was 8.28 SD 4.93 in the group of all preterm children and 6.47 SD 3.97 in the group of all controls tested in one session. There was no significant difference between groups and blocks and no significant group by block interaction (all *P* values > 0.13). Mean total number of alpha responses count was 8.28 (SD 4.93) and 6.47 (SD 3.97) in the first session, and 4.58 (SD 3.8) and 3.83 (SD 2.66) in the second session in the preterm and control groups, respectively. There was no significant difference between groups, blocks, sessions and no significant interaction effects (all *P* values > 0.1).

UR onset and peak time in the unpaired US-only trials occurred numerically later in the group of all preterm children compared to controls (Table 2). UR peak time was significantly different between groups (*P* = 0.007). UR onset and duration were not significantly different between groups (*P* values = 0.06). In the subgroup of children tested twice, UR onset appeared delayed in preterm children in session 1, but within the range of the controls in session 2. UR onset was later in preterms than in controls, but only on a trend level [F (1, 22) = 6.0, *P* = 0.02]. Session effect and session by group interactions were not significant (*P* > 0.32). Considering UR peak time and duration group, session or session by group (interaction) effects were not significant (*P* values > 0.07).

Considering the group of all preterm and control children, comparison of spontaneous blink rate showed no significant group difference, no difference between the beginning and end of the session and no significant interaction (all *P* values > 0.084). The group difference in the subgroups tested twice reached significance [F(1 22) = 4.32, *P* = 0.049] with mean spontaneous blink rate being higher in preterm children compared to controls. In both groups spontaneous blink rate was lower in the second session compared to the first [session effect: F (1, 22) = 23.23, *P* = 0.001]. There were no significant differences between the beginning and end of the session and no significant interactions (see Supplementary Table 2 for details). As expected, spontaneous blink rate was less in children compared to adults cf. mean values in Table 3;^46,47^.

#### Correlation with birth weight, gestational age and ataxia scores

In the group of all preterm children total CR incidence showed no significant correlation with birth weight (ρ = −0.09, *P* = 0.61), gestational age in days (ρ = −0.11, *P* = 0.53), ICARS score (ρ = −0.12, *P* = 0.51) or SARA score (ρ = −0.12, *P* = 0.5). Likewise, no significant correlations were found in the subgroup of preterm children tested twice, neither in the first nor in the second session (R = −0.44–0.47, *P* = 0.12–0.96).

A significant negative correlation was found comparing birth weight and ICARS score (R = −0.35, *P* = 0.05) and gestational age in days and ICARS score (ρ = −0.39, *P* = 0.03) in the group of all preterm children and in the subgroup of preterm children tested twice (R = −0.621, *P* = 0.03 and R = −0.64, *P* = 0.03, respectively). That is a lower birth weight and reduced gestational age were associated with a higher ataxia score.

## Discussion

Acquisition of conditioned eyeblink responses was reduced in very preterm born preschool children and very preterm born young adults (<32 weeks gestation). Findings are consistent with the assumption that prematurity results in cerebellar dysfunction. Because focal cerebellar lesions were excluded in the preterm population, the present findings are best explained by impaired cerebellar development. The present findings suggest that detrimental effects on the functional integrity of the cerebellum are long lasting and present at least until early adulthood. Based on the present findings alone, however, we cannot exclude that extra-cerebellar pathology played an additional role, which will be discussed in detail below.

Major events in cerebellar development take place at the passage from the second to the third trimester of pregnancy^14,48^. Sonic hedgehog secreted by Purkinje cells induces high proliferation in the outer half of the external granular layer of the cerebellar cortex^49^. From 30 to 40 weeks gestation cerebellar growth and foliation is dominated by proliferation of granule precursor cells and their migration to the internal granule layer of the cerebellar cortex^10,13,50^. At the same time cerebellar nuclei develop and interconnect with the cerebellar cortex^51^. During this critical period of high proliferation and migration in the cerebellar cortex, preterm infants are born into an unnatural environment, often being critically ill and exposed to potential harmful agents and procedures, putting them at high risk for developmental abnormalities^52–54^. As outlined in the introduction, brain MRI studies indeed show that the cerebellar volume is reduced in preterm infants even in the absence of focal cerebellar lesions^11,19,55,56^. Haldipur *et al*.^56^ describe abnormalities in the thickness and density of the granular cell layer in very preterm children without focal cerebellar lesions. Granular cells are an important part of the cerebellar circuit underlying eyeblink conditioning^32,57,58^. The best-known cerebellar model of eyeblink conditioning implies that the conditioned stimulus (CS) is conveyed to the granular cell layer via the pontine nuclei and mossy fibres. Granule cells transmit the CS signals via parallel fibres to the Purkinje cells. Purkinje cells receive unconditioned stimulus (US) information via the inferior olive and climbing fibre system. Based on this model, after repeated CS-US stimulation, Purkinje cells learn to pause to the CS, which results in less inhibition of the cerebellar nuclei, allowing conditioned responses to occur^58^. More recent studies found that granule cells not only transmit CS information to the Purkinje cells, but that learning related plastic changes occur also at the level of the granule cells^31,59^. Thus, developmental abnormalities in the granular cell layer within the cerebellar cortex are well suited to explain disordered eyeblink conditioning in preterm subjects. Studies in patients with focal cerebellar disease show that lobule VI with extension into Crus I are the cerebellar cortical areas, which are critically involved in eyeblink conditioning in humans^34,35^. It would be of interest to perform voxel-based morphometry (VBM) MRI studies in preterm subjects in the future, to investigate whether reduced eyeblink conditioning is correlated with reduced grey matter volume in lobules VI/Crus I.

The interposed nuclei, inferior olive and pontine nuclei are also essential parts of the cerebellar circuitry underlying eyeblink conditioning^60,61^. Neuronal loss in the dentate nuclei and inferior olive has been reported in preterm children without white matter abnormalities at autopsy^62^. Thus, abnormalities of multiple hubs of the cerebellar learning circuitry may contribute to the observed reduction of learning.

Eyeblink conditioning was reduced in both, preterm born adults and preschool children compared to age-matched controls. Whereas significant deficits in preterm adults were present in a single session, deficits in preschool preterm children were most prominent in a second session. Control children, but not preterm children, showed significantly higher CR incidences already in the first block of session 2. Although retention likely played a role^63^, function appeared to improve less between sessions in preterm children compared to controls. Irrespective of the consequences of prematurity, there were significant age effects. Control children at the age of 5–6 years needed more sessions and acquired less conditioned responses than young control adults. Furthermore, conditioned responses occurred earlier in children than in adults, thus they were less well timed^64,65^. Age-dependency of eyeblink conditioning is well known in the animal and human literature^66–68^. In a recent study, Löwgren *et al*.^69^ found that acquisition of conditioned eyeblink responses attains adult levels in humans at the age of 9 years. Similar to eyeblink conditioning, ataxia rating scales show an age-dependency^43,44^. Neurological examination showed mild balance deficits and limb incoordination in control children, which resulted unsurprisingly in higher ataxia rating scores than in adults. Scores on the International Cooperative Ataxia Rating Scale (ICARS,^41^ and Scale of Assessment and Rating of Ataxia (SARA;^42^ are increased in children up to the age of 8–12 years^43,44^. A limitation of our study is the lack of brain MRI in preterm and control children at the time of the testing, as potential lesions or developmental disturbances may confound results. Age-dependency, however, is likely explained by on-going development and maturation of the cerebellar circuits. Regarding eyeblink conditioning, developmental changes have been reported in both sensory pathways (CS and US) as well as in the inhibitory feedback loop of the cerebellar nuclei to the US pathway^67^.

In healthy subjects, the significant increase of the number of conditioned responses during learning is accompanied by a temporal shift of the response towards the onset of the unconditioned stimulus (US) and an increase in response size^64,65,67^. This was observed in the present study in control children and adults, but not in preterm children. The control of timing and size of conditioned responses is also cerebellar-dependent^70,71^. Conditioned responses occur significantly earlier in patients with cerebellar disease^72^. In contrast to preterm children, in preterm born adults learning dependent changes in timing and size was generally preserved, except for a lesser increase of response duration compared to controls. Performance-related, but not learning-related, deficits may be ameliorated during maturation. Likewise, preterm children but not preterm born adults showed abnormal ataxia rating scores. Note, that total SARA scores of 3 or more are considered abnormal in adults^42^. None of the preterm born adults had total SARA score higher than 2.

It is important to state that in prematurity, brain lesions are not restricted to the cerebellum. Involvement of the brainstem has been reported in histological, MRI and electrophysiological data in preterm infants even in the absence of focal lesions^62,73,74^. To exclude that accompanying lesions of the afferent and efferent blink reflex pathways within the brainstem played a role, unconditioned eyeblink responses (UR) were examined in explicitly unpaired trials^75^. In preterm born adults, unconditioned responses were not different from controls. In preterm children, however, URs were slightly delayed. Therefore, it cannot be excluded that accompanying (maturational) deficits in blink reflex pathways contributed to reduced eyeblink conditioning in preterm children.

In addition to the cerebellum, however, developmental alterations of the preterm brain predominantly occur above the brainstem level^52,54^. This cannot be excluded in the present preterm population, although cerebral volumes in the adult preterm group did not show a significant difference compared to controls. In the present study short delay eyeblink conditioning was tested, the most simple form of associative motor learning. In contrast to more complex forms of conditioning, for example trace eyeblink conditioning; cerebral areas are not essential for delay conditioning^76,77^. Supratentorial areas, however, have been found to play a modulatory role in delay conditioning. Abnormal functioning or lesions of the hippocampus and amygdala have been shown to slow down acquisition^78,79^. In preterm infants with favourable outcome and lack of focal and obvious white matter lesions neuroimaging studies have revealed volume reductions not only in the cerebellum but also in cerebral areas including the amygdala and hippocampus^80–82^. Thus, it cannot be excluded that supratentorial lesions may have contributed to slowed acquisition, which was observed in preterm born adults.

Extinction of conditioned responses was preserved in preterm subjects. Comparable results of impaired acquisition but preserved extinction were found in patients with essential tremor^36^ and migraine^37^. In patients with cerebellar degeneration and focal disease extinction was diminished^83^. However, because CR acquisition is severely reduced in this patient population, interpretation of extinction data is difficult (see ref.^35^ for discussion). Extinction of conditioned eyeblinks is thought to be a combination of unlearning and newly learned inhibition^84–86^. Whereas unlearning may primarily take place in the cerebellar cortex^87,88^, prefrontal areas and hippocampus are thought to be key regions in learned inhibition. It has been proposed that learned inhibition is mediated to the cerebellum via the amygdala^85^. Preserved extinction suggests that cerebral regions involved in learned inhibition were likely preserved in preterm children.

Our data are in accordance with the notion that very preterm birth results in long-term detrimental effects on the functional integrity of the cerebellum. Functional implications of cerebellar developmental disorders likely contribute not only to motor but also to cognitive impairments^15,89^. Cognitive, emotional and behavioural deficits become evident as the child grows up and functional demands increase^90,91^. The cerebellum has known anatomical connections with prefrontal and limbic areas^92,93^. Development of the connected cerebral areas likely depends on a fully functioning cerebellum^94,95^, a concept that is described with the term “developmental diaschisis”^96,97^. Reduced development of brain areas connected to the cerebellum has been suggested to be a consequence of disrupted cerebellar learning mechanisms^98^. Our finding of reduced associative learning provides support for this assumption.

The identification of children at risk for neurodevelopmental impairment is necessary as early as possible to promote the child to develop according to its full capacity. Reeb-Sutherland and Fox have proposed eyeblink conditioning as a non-invasive biomarker for neurodevelopmental disorders^99^. Eyeblink conditioning is feasible in human newborns^27,100^, even during natural sleep^101,102^. Future studies need to explore whether eyeblink conditioning is a useful screening tool for subclinical cerebellar dysfunction in the preterm population.

In conclusion, the present data are in accordance with the assumption that preterm birth impedes function of the cerebellum even in the absence of focal cerebellar lesions. Cerebellar dysfunction seems to be present in childhood and persists at least into early adulthood. Future studies, however, are needed to further explore the relative contribution of cerebellar and extracerebellar pathology on eyeblink conditioning in preterm infants.

## Materials and Methods

### Study population

A group of preterm born young adults and a group of preterm preschool children were compared to corresponding age- and sex-matched control groups. Preterm participants and term born controls were enrolled between April 2015 and June 2016. Records were screened from all preterm subjects born in 1997 (adult group) and 2009/2010 (children group) in our tertiary care hospital in Essen, Germany. From 82 surviving infants in 1997, 64 met the inclusion criteria and were contacted. 50 were lost for follow up/did not reply, 2 refused to be involved. Thus, 12 remained (30%) who participated. The additional preterm adult participants were enrolled via social media and advertisement on the hospital homepage and newsletter. Control subjects were either peers of preterm participants or recruited by advertisement. Three adult controls had to be excluded because of drug abuse or incidental findings on MRI.

In the preterm children’s group, from 95 surviving children in 2009/2010, 34 (36%) could be enrolled. 21 were lost for follow up, 30 refused participation and 10 did not meet inclusion criteria. There were 2 drops outs.

**Figure 5:**
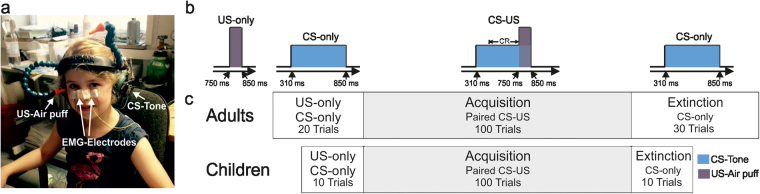
Experimental set-up (**a**) and delay eyeblink conditioning paradigm (**b,c**). The number of trials in the unpaired, acquisition and extinction phases is shown for the adult and the children’s group, respectively. CS = conditioned stimulus (that is, a tone of 540 ms duration indicated in blue); US = unconditioned stimulus (that is, an air puff of 100 ms duration, indicated in grey). For more details see text.

The adult group consisted of 20 very preterm born young adults [10 male, 10 female; mean age 19.4 years (yrs) (standard deviation (SD) 1.6), range 18.1–24.5 yrs; mean birth weight 1219 g (SD 424), range 650–1970 g] and 20 healthy young adult controls [10 male, 10 female; mean age 19.9 yrs (SD 1.6), range 18.5–23.5 yrs; mean birth weight 3471 g (SD 476), range 2540–4300 g]. The group of children consisted of 32 children born at <32 weeks gestation at preschool age [16 male, 16 female, mean age 5.8 yrs (SD 0.58), range 5.0–6.6 yrs; mean birth weight 1047 g (SD 322), range 540–1670 g] and 32 healthy controls [16 male, 16 female; mean age 5.8 yrs (SD 0.58), range 5.0–6.7 yrs; mean birth weight 3397 g (SD 376), range 2630–4060 g]. All control participants were term born > 37 weeks gestational age. Perinatal characteristics are given in Tables 1 and 2. In very preterm children extracerebellar lesions [i.e., intraventricular hemorrhage (IVH) > II°, cystic periventricular leukomalacia (PVL), punctate white matter lesions > 4 cluster per side] and focal cerebellar lesions were excluded based on 1.5 T brain magnetic resonance images (MRI) performed at term equivalent (neonatal) age. In young adults, extracerebellar lesions and focal cerebellar lesions were excluded based on brain 3 T MRIs acquired at the time of testing as part of another study. In addition, IVH > II°, cystic PVL and focal cerebellar lesions were excluded based on postnatal ultrasound reports. Brain MRI at term equivalent age was not part of the diagnostic routine at the time the young adults were born. All MRI scans were examined by two experienced pediatric radiologists (B.S. and S.S.). None of the preterm or the control participants received any medication modifying nervous system functions or had a history of drug- or alcohol abuse. None had a history of chromosomal alterations. All participants were attending or had finished a regular kindergarten, preschool or school. None of the participants were treated for attention deficit hyperactivity disorder (ADHD) or any other psychiatric disorder. All participants received a complete neurological examination at the day of the testing. Adults were examined by B.M.H. and children by L.T., both experienced paediatricians. Possible signs of cerebellar ataxia were rated based on the ICARS^41^ and the SARA^42^. Clinical data for preterm and control groups as well as brain MRI and ultrasound findings in the preterm groups are summarized in Tables 1 and 3. The study was approved by the local ethical committee of the University Hospital Essen (15-6181-BO and 10-4427-BO). The experiment and examinations were performed in accordance with relevant guidelines and regulations. All subjects and parents or legal guardians of minors gave informed written consent prior to participation. The parents of the participant whose photograph was used in Fig. 5 gave a signed statement of informed consent to publish the image in an online open access publication. The family had the opportunity to see the manuscript prior to submission.

### Experimental design

Eyeblink conditioning was performed using a standard delay paradigm introduced by^103^. In delay conditioning the CS precedes the US by a fixed time interval and co-terminates with the US (Fig. 5b). The experimental set-up was the same as in previous experiments of our group^34,35,72^. Participants sat comfortably on a chair with their eyes open. They wore a helmet with a nozzle and headphones (Fig. 5a). The air puff-US was provided through the nozzle with duration of 100 ms and intensity of 4 Bar at source. The nozzle was directed to the outer canthus of the right eye at a distance of approximately 10 mm. The tone-CS [1000 Hz, 70 dB sound pressure level (SPL) at source, duration 540 ms] was presented to the right ear via headphones. It was superimposed on a continuous white noise of 60 dB sound pressure level (SPL) applied bilaterally to mask environmental noise.

Surface electromyography (EMG) recordings were taken from the orbicularis oculi muscles bilaterally. The overlying skin was cleaned with mildly abrasive paste. Gold cup electrodes (10 mm diameter) were fixed below the lower eyelids and on the nasion bilaterally. Signals were fed to EMG amplifiers (sampling rate 1000 Hz, band pass filter between 100 Hz and 2 kHz), full wave rectified and further filtered offline (100 Hz). Data was collected for 2000 ms per trial. Data collection started 310 ms prior CS onset (5b).

In the adult group the session started with ten US-only and ten CS-only trials in an unpaired pseudorandom order, followed by an acquisition phase of 100 paired CS-US trials. The session ended with an extinction phase of 30 CS-only trials (Fig. 5c). In the children’s group five US-only and five CS-only trials were presented in an unpaired pseudorandom order, followed by 100 paired CS-US acquisition trials and ten CS-only extinction trials (Fig. 5c). The inter-trial interval pseudo-randomly varied between 20 to 35 seconds. Spontaneous blinks were recorded for one minute at the beginning and end of the session.

In order to maintain vigilance and attention a silent movie (appropriate for adults, e.g. Mr. Bean by Tiger Aspect Productions, and children, e.g. Shaun the Sheep by Aardman Animations) was shown using a DVD player. Participants were informed beforehand about the air puff delivered to their right eye and the tone via headphones. The neurophysiological background and essentials of classical conditioning were not explained.

All participants were tested in one session, which lasted 70 minutes in adults and 60 minutes in children. Subgroups of twelve preterm preschool children [6 male, 6 female, mean age 6.1 yrs (SD 0.35), range 5.7–6.8 yrs] and of twelve matched healthy children [6 male, 6 female, mean age 6.0 yrs (SD 0.68), range 5.2–7.0 yrs] were tested twice. The time interval between the two sessions was three months [preterm group: mean 89.5 days (SD 6.0), range 75–97 days; control group: mean 82.2 days (SD 5.2), range 77–91 days].

### Data analysis

EMG recordings were analysed semi-automatically on a trial-by-trial basis using a custom made software^104^. Conditioned responses (CRs) were automatically identified within the CS-US window. Responses occurring within the 150 ms interval after CS onset were defined as alpha responses (that is, reflexive responses to the tone) and not as CRs^105^. CRs in trials with a spontaneous blink prior to CS onset were not included in statistical analysis^106^. CRs were identified (and CR onset defined) when EMG activity reached 7.5% of the EMG maximum in each trial with a minimum duration of 20 ms. All trials were visually inspected and implausible identification of CRs was manually corrected. A technician, blinded to the subjects’ group, performed data analysis.

The total number of acquisition and extinction trials recorded was subdivided into blocks of ten trials each. The number of conditioned responses (CR) was expressed as the percentage of trials containing CRs with respect to each block of ten trials (percentage CR incidence) and the total number of trials (total percentage CR incidence).

In addition to CR onset, CR peak time, CR duration and CR area were quantified. CR peak time was defined at the time of maximum amplitude before US onset. CR duration was defined as the time interval between CR onset and return of EMG activity to 7.5% of the EMG maximum prior US onset. In case CR and unconditioned response (UR) overlapped (which is often the case in paired CS-US trials), CR duration was defined as the time interval between CR onset and US onset. CR area was assessed in a fixed time interval of 50 ms after CR onset (50 ms integral). Baseline area was assessed in an interval of 100 ms prior US onset in each trial. Baseline area /2 was subtracted from the CR- 50 ms integral. To allow for group comparisons in surface EMG recordings, the mean CR area across all CRs in each individual subject was set as 100% and each individual CR area expressed as % of the mean. This allowed group comparisons of changes in CR area across time. Absolute CR area values were not compared.

In unpaired US-only trials, onset, peak time and duration of the UR were assessed using the same custom made software. The numbers of spontaneous blinks were determined based on visual inspection of the one-minute recordings at the beginning and end of each session.

### Statistical analysis

Statistical analyses were performed separately in children and adults using SPSS software (Version 19.0, IBM Corp., Armonk, NY). CR incidence was the primary outcome measure. To assess acquisition (that is learning), CR incidences were compared across the ten acquisition blocks between preterm and control subjects. Analyses of variance (ANOVA) with repeated measures were calculated with CR incidence as dependent variable, block (1–10) as within subject-factor, and group (preterm group vs. control group) as between-subject factor. In the subgroups of children undergoing second testing, session (session 1 vs. session 2) was used as additional within-subject factor.

To assess extinction (that is unlearning), CR incidences were compared between the last acquisition block and the three extinction blocks in adults, and between the last acquisition block and the one extinction block in children. ANOVA with repeated measures were calculated in the same way as for acquisition with the exception of the within-subject factor block, which was four in adults and two in children.

*P* values for effects were set at < 0.05. Greenhouse-Geisser correction was applied where appropriate and degrees of freedom adjusted accordingly.

In the acquisition phase, ANOVA with repeated measures were used to compare CR timing and performance parameters (i.e., CR onset, peak time, duration, and area). For each parameter and subject, mean values were calculated for 20 consecutive trials. Mean values were used as dependent variable, block (1–5) as within subject-factor, and group (preterm group vs. control group) as the between-subject factor. In the subgroup of children tested twice, session (session 1 vs. session 2) was used as additional within-subject factor. Due to the small number of extinction trials and hence a low reliability, no statistical comparison of CR timing and performance parameters was performed in extinction trials. *P* values were set as < 0.0125 (Bonferroni correction).

Alpha response count was compared between groups and acquisition blocks analogous to the statistical analysis of CR incidence. Spontaneous blink rates were compared between groups using ANOVA with repeated measures. The assessments of the beginning and end of each session were used as within-subject factor. *P* values for effects were set at < 0.05. Greenhouse-Geisser correction was applied where appropriate and degrees of freedom adjusted accordingly.

In adults and the group of all children, mean UR onset, peak time and duration were compared between preterm and control groups. Unpaired t test was used in normally distributed data and Wilcoxon rank sum test in non-normally distributed data. UR parameters in the children tested twice were compared between groups using ANOVA with repeated measures. The assessments of the two sessions were used as within-subject factor. *P* values were set as < 0.016 (Bonferroni correction).

Parameters, which showed significant differences between groups, were correlated with birth weight, gestational, age in days and total ICARS and SARA scores in preterm participants. Pearson correlation analysis was used in normally distributed data and Spearman correlation analysis in non-normally distributed data. *P* values for effects were set at < 0.05.

### Data availability

The datasets analysed during the current study are available from the corresponding author on reasonable request.

## Electronic supplementary material

Supplementary Material

## References

[1] Howson CP, Kinney MV, McDougall L, Lawn JE, Born Too Soon Preterm Birth Action Group (2013). Born too soon: Preterm birth matters. Reprod Health.

[2] Saigal S, Doyle LW (2008). An overview of mortality and sequelae of preterm birth from infancy to adulthood. Lancet.

[3] Platt MJ (2007). Trends in cerebral palsy among infants of very low birthweight (<1500 g) or born prematurely (<32 weeks) in 16 european centres: A database study. Lancet.

[4] Bhutta AT, Cleves MA, Casey PH, Cradock MM, Anand KJ (2002). Cognitive and behavioral outcomes of school-aged children who were born preterm: A meta-analysis. JAMA.

[5] Marlow N, Wolke D, Bracewell MA, Samara M (2005). Neurologic and developmental disability at six years of age after extremely preterm birth. N Engl J Med.

[6] Lohaugen GC (2010). Cognitive profile in young adults born preterm at very low birthweight. Dev Med Child Neurol.

[7] Volpe JJ (2009). The encephalopathy of prematurity–brain injury and impaired brain development inextricably intertwined. Semin Pediatr Neurol.

[8] Pierson CR, Al Sufiani F (2016). Preterm birth and cerebellar neuropathology. Semin Fetal Neonatal Med.

[9] Limperopoulos C (2005). Cerebellar hemorrhage in the preterm infant: Ultrasonographic findings and risk factors. Pediatrics.

[10] Volpe JJ (2009). Cerebellum of the premature infant: Rapidly developing, vulnerable, clinically important. J Child Neurol.

[11] Messerschmidt A (2005). Disruption of cerebellar development: Potential complication of extreme prematurity. AJNR Am J Neuroradiol.

[12] Limperopoulos C (2005). Late gestation cerebellar growth is rapid and impeded by premature birth. Pediatrics.

[13] Lemire, R., Loeser J, Leech R, Alvrod, EJ. *Normal and abnormal development of the human nervous system*. (Harper & Row, 1975).

[14] Dobbing J (1974). The later growth of the brain and its vulnerability. Pediatrics.

[15] Limperopoulos C (2007). Does cerebellar injury in premature infants contribute to the high prevalence of long-term cognitive, learning, and behavioral disability in survivors?. Pediatrics.

[16] Steinlin M (2008). Cerebellar disorders in childhood: Cognitive problems. Cerebellum.

[17] Schmahmann JD, Sherman JC (1998). The cerebellar cognitive affective syndrome. Brain.

[18] Keunen K (2016). Brain volumes at term-equivalent age in preterm infants: Imaging biomarkers for neurodevelopmental outcome through early school age. J Pediatr.

[19] Allin M (2001). Cognitive and motor function and the size of the cerebellum in adolescents born very pre-term. Brain.

[20] Allin MP (2005). Vermis and lateral lobes of the cerebellum in adolescents born very preterm. Neuroreport.

[21] Murray AL (2014). Neonatal brain pathology predicts adverse attention and processing speed outcomes in very preterm and/or very low birth weight children. Neuropsychology.

[22] Omizzolo C (2014). Neonatal brain abnormalities and memory and learning outcomes at 7 years in children born very preterm. Memory.

[23] Lind A (2010). Relations between brain volumes, neuropsychological assessment and parental questionnaire in prematurely born children. Eur Child Adolesc Psychiatry.

[24] Spittle AJ (2010). Reduced cerebellar diameter in very preterm infants with abnormal general movements. Early Hum Dev.

[25] Messerschmidt A (2008). Disrupted cerebellar development in preterm infants is associated with impaired neurodevelopmental outcome. Eur J Pediatr.

[26] Levisohn L, Cronin-Golomb A, Schmahmann JD (2000). Neuropsychological consequences of cerebellar tumour resection in children: Cerebellar cognitive affective syndrome in a paediatric population. Brain.

[27] Woodruff-Pak, D. S. & Steinmetz, J. E. in *Eye**blink classical**conditioning: Volume I. Applications in humans*. (eds Woodruff-Pak, D. S. & Steinmetz, J. E.) 1–17 (Kluwer, 2000).

[28] Yeo CH, Hardiman MJ, Glickstein M (1984). Discrete lesions of the cerebellar cortex abolish the classically conditioned nictitating membrane response of the rabbit. Behav Brain Res.

[29] Mauk MD, Li W, Khilkevich A, Halverson H (2014). Cerebellar mechanisms of learning and plasticity revealed by delay eyelid conditioning. Int Rev Neurobiol.

[30] McCormick DA, Thompson RF (1984). Neuronal responses of the rabbit cerebellum during acquisition and performance of a classically conditioned nictitating membrane-eyelid response. J Neurosci.

[31] Gao Z, van Beugen BJ, De Zeeuw CI (2012). Distributed synergistic plasticity and cerebellar learning. Nat Rev Neurosci.

[32] Bracha V (2004). Role of the cerebellum in eyeblink conditioning. Prog Brain Res.

[33] Daum I (1993). Classical conditioning after cerebellar lesions in humans. Behav Neurosci.

[34] Gerwig M (2003). Comparison of eyeblink conditioning in patients with superior and posterior inferior cerebellar lesions. Brain.

[35] Ernst TM (2016). Pronounced reduction of acquisition of conditioned eyeblink responses in young adults with focal cerebellar lesions impedes conclusions on the role of the cerebellum in extinction and savings. Neuropsychologia.

[36] Kronenbuerger M, Gerwig M, Brol B, Block F, Timmann D (2007). Eyeblink conditioning is impaired in subjects with essential tremor. Brain.

[37] Gerwig M, Rauschen L, Gaul C, Katsarava Z, Timmann D (2014). Subclinical cerebellar dysfunction in patients with migraine: Evidence from eyeblink conditioning. Cephalalgia.

[38] Frings M (2010). Timing of conditioned eyeblink responses is impaired in children with attention-deficit/hyperactivity disorder. Exp Brain Res.

[39] Nicolson RI, Daum I, Schugens MM, Fawcett AJ, Schulz A (2002). Eyeblink conditioning indicates cerebellar abnormality in dyslexia. Exp Brain Res.

[40] Coffin JM, Baroody S, Schneider K, O’Neill J (2005). Impaired cerebellar learning in children with prenatal alcohol exposure: A comparative study of eyeblink conditioning in children with ADHD and dyslexia. Cortex.

[41] Trouillas P (1997). International cooperative ataxia rating scale for pharmacological assessment of the cerebellar syndrome. The ataxia neuropharmacology committee of the world federation of neurology. J Neurol Sci.

[42] Schmitz-Hübsch T (2006). Scale for the assessment and rating of ataxia: Development of a new clinical scale. Neurology.

[43] Brandsma R (2014). Ataxia rating scales are age-dependent in healthy children. Dev Med Child Neurol.

[44] Sival DA, Brunt ER (2009). The international cooperative ataxia rating scale shows strong age-dependency in children. Dev Med Child Neurol.

[45] Beyer L, Batsikadze G, Timmann D, Gerwig M (2017). Cerebellar tdcs effects on conditioned eyeblinks using different electrode placements and stimulation protocols. Front Hum Neurosci.

[46] Zametkin AJ, Stevens JR, Pittman R (1979). Ontogeny of spontaneous blinking and of habituation of the blink reflex. Ann Neurol.

[47] Lavezzo MM, Schellini SA, Padovani CR, Hirai FE (2008). Eye blink in newborn and preschool-age children. Acta Ophthalmol.

[48] Sidman RL, Rakic P (1973). Neuronal migration, with special reference to developing human brain: A review. Brain Res.

[49] Carletti B, Rossi F (2008). Neurogenesis in the cerebellum. Neuroscientist.

[50] Nowakowska-Kotas M, Kedzia A, Dudek K (2014). Development of external surfaces of human cerebellar lobes in the fetal period. Cerebellum.

[51] Elsen, G. E., Juric-Sekhar, G., Daza, R. A. M. & Hevner, R. F. in *Handbook of the cerebellum and cerebellar disorders*. (eds Manto, M. *et al*.),179–205 (Springer, 2013).

[52] Ortinau C, Neil J (2015). The neuroanatomy of prematurity: Normal brain development and the impact of preterm birth. Clin Anat.

[53] Anderson PJ, Cheong JL, Thompson DK (2015). The predictive validity of neonatal mri for neurodevelopmental outcome in very preterm children. Semin Perinatol.

[54] Volpe JJ (2009). Brain injury in premature infants: A complex amalgam of destructive and developmental disturbances. Lancet Neurol.

[55] Tam EW (2013). Potential mechanisms of cerebellar hypoplasia in prematurity. Neuroradiology.

[56] Haldipur P (2011). Preterm delivery disrupts the developmental program of the cerebellum. PLoS One.

[57] Christian KM, Thompson RF (2003). Neural substrates of eyeblink conditioning: Acquisition and retention. Learn Mem.

[58] Linden DJ (2003). From molecules to memory in the cerebellum. Science.

[59] Giovannucci A (2017). Cerebellar granule cells acquire a widespread predictive feedback signal during motor learning. Nat Neurosci.

[60] Yeo CH, Hardiman MJ, Glickstein M (1985). Classical conditioning of the nictitating membrane response of the rabbit. Ii. Lesions of the cerebellar cortex. Exp Brain Res.

[61] McCormick DA, Thompson RF (1984). Cerebellum: Essential involvement in the classically conditioned eyelid response. Science.

[62] Pierson CR (2007). Gray matter injury associated with periventricular leukomalacia in the premature infant. Acta Neuropathol.

[63] Timmann D, Gerwig M, Frings M, Maschke M, Kolb FP (2005). Eyeblink conditioning in patients with hereditary ataxia: A one-year follow-up study. Exp Brain Res.

[64] Boneau CA (1958). The interstimulus interval and the latency of the conditioned eyelid response. J Exp Psychol.

[65] Prokasy WF, Ebel HC, Thompson DD (1963). Response shaping at long interstimulus intervals in classical eyelid conditioning. J Exp Psychol.

[66] Stanton ME, Freeman JH, Skelton RW (1992). Eyeblink conditioning in the developing rat. Behav Neurosci.

[67] Freeman JH (2014). The ontogeny of associative cerebellar learning. Int Rev Neurobiol.

[68] Cheng DT (2014). Functional MRI of cerebellar activity during eyeblink classical conditioning in children and adults. Hum Brain Mapp.

[69] Löwgren K (2017). Performance in eyeblink conditioning is age and sex dependent. PLoS One.

[70] Perrett SP, Ruiz BP, Mauk MD (1993). Cerebellar cortex lesions disrupt learning-dependent timing of conditioned eyelid responses. J Neurosci.

[71] Welsh JP, Harvey JA (1989). Cerebellar lesions and the nictitating membrane reflex: Performance deficits of the conditioned and unconditioned response. J Neurosci.

[72] Gerwig M (2005). Timing of conditioned eyeblink responses is impaired in cerebellar patients. J Neurosci.

[73] Makropoulos A (2016). Regional growth and atlasing of the developing human brain. Neuroimage.

[74] Stipdonk LW (2016). Auditory brainstem maturation in normal-hearing infants born preterm: A meta-analysis. Dev Med Child Neurol.

[75] Topka H, Valls-Sole J, Massaquoi SG, Hallett M (1993). Deficit in classical conditioning in patients with cerebellar degeneration. Brain.

[76] Thompson RF, Steinmetz JE (2009). The role of the cerebellum in classical conditioning of discrete behavioral responses. Neuroscience.

[77] Hesslow, G. in *Neural control of movement*. (eds Ferrell, W.R. & Proske, U.) 117–122. (Plenum Press., 1995).

[78] Woodruff-Pak DS, Li YT, Hinchliffe RM, Port RL (1997). Hippocampus in delay eyeblink classical conditioning: Essential for nefiracetam amelioration of learning in older rabbits. Brain Res.

[79] Yang Y, Lei C, Feng H, Sui JF (2015). The neural circuitry and molecular mechanisms underlying delay and trace eyeblink conditioning in mice. Behav Brain Res.

[80] Ball G (2012). The effect of preterm birth on thalamic and cortical development. Cereb Cortex.

[81] Cismaru AL (2016). Altered amygdala development and fear processing in prematurely born infants. Front Neuroanat.

[82] Peterson BS (2000). Regional brain volume abnormalities and long-term cognitive outcome in preterm infants. JAMA.

[83] Gerwig M (2006). Extinction of conditioned eyeblink responses in patients with cerebellar disorders. Neurosci Lett.

[84] Medina JF, Nores WL, Mauk MD (2002). Inhibition of climbing fibres is a signal for the extinction of conditioned eyelid responses. Nature.

[85] Hu C, Zhang LB, Chen H, Xiong Y, Hu B (2015). Neurosubstrates and mechanisms underlying the extinction of associative motor memory. Neurobiol Learn Mem.

[86] Robleto K, Poulos AM, Thompson RF (2004). Brain mechanisms of extinction of the classically conditioned eyeblink response. Learn Mem.

[87] Jirenhed DA, Bengtsson F, Hesslow G (2007). Acquisition, extinction, and reacquisition of a cerebellar cortical memory trace. J Neurosci.

[88] Medina JF, Garcia KS, Mauk MD (2001). A mechanism for savings in the cerebellum. J Neurosci.

[89] Tavano A (2007). Disorders of cognitive and affective development in cerebellar malformations. Brain.

[90] Doyle LW (2014). Long term follow up of high risk children: Who, why and how?. BMC Pediatr.

[91] Keunen K (2012). Brain tissue volumes in preterm infants: Prematurity, perinatal risk factors and neurodevelopmental outcome: A systematic review. J Matern Fetal Neonatal Med.

[92] Blatt, G. J., Oblak, A. L. & Schmahmann, J. D. in *Handbook of the cerebellum and cerebellar disorders*. (eds Manto, M. *et al*.) 479–496 (Springer, 2013).

[93] Strick PL, Dum RP, Fiez JA (2009). Cerebellum and nonmotor function. Annu Rev Neurosci.

[94] Bolduc ME (2011). Cerebellar malformations alter regional cerebral development. Dev Med Child Neurol.

[95] Wang SS, Kloth AD, Badura A (2014). The cerebellum, sensitive periods, and autism. Neuron.

[96] Brown-Séquard C (1875). On the hereditary transmission of effects of certain injuries to the nervous system. The Lancet.

[97] von Monakow, C. *Die Lokalisation im Großhirn und der Abbau der Funktion durch kortikale Herde*. (Bergmann, JF 1914).

[98] Stoodley CJ, Limperopoulos C (2016). Structure-function relationships in the developing cerebellum: Evidence from early-life cerebellar injury and neurodevelopmental disorders. Semin Fetal Neonatal Med.

[99] Reeb-Sutherland BC, Fox NA (2015). Eyeblink conditioning: A non-invasive biomarker for neurodevelopmental disorders. J Autism Dev Disord.

[100] Wenger, M. A. An investigation of conditioned responses in human infants. *University of Iowa Studies: Child Welfare*. (1936).

[101] Tarullo AR (2016). Neonatal eyelid conditioning during sleep. Dev Psychobiol.

[102] Fifer WP (2010). Newborn infants learn during sleep. Proc Natl Acad Sci USA.

[103] Gormezano, I. & Kehoe, E. J. In *Handbook of learning** and cognitive processes, conditioning and behavior theory*. (ed Estes, W. K.) 143–179 (Lawrence Erlbaum, 1975).

[104] Gerwig M (2010). Evaluation of multiple-session delay eyeblink conditioning comparing patients with focal cerebellar lesions and cerebellar degeneration. Behav Brain Res.

[105] Woodruff-Pak DS, Papka M, Ivry RB (1996). Cerebellar involvement in eyeblink classical conditioning in humans. Neuropsychology.

[106] Bracha V, Zhao L, Wunderlich DA, Morrissy SJ, Bloedel JR (1997). Patients with cerebellar lesions cannot acquire but are able to retain conditioned eyeblink reflexes. Brain.

[107] Apgar V (1953). A proposal for a new method of evaluation of the newborn infant. Curr Res Anesth Analg.

[108] Aukland SM (2008). Assessing ventricular size: Is subjective evaluation accurate enough? New mri-based normative standards for 19-year-olds. Neuroradiology.

[109] Brandauer B (2008). Impairments of prehension kinematics and grasping forces in patients with cerebellar degeneration and the relationship to cerebellar atrophy. Clin Neurophysiol.

[110] Nguyen The Tich S (2009). A novel quantitative simple brain metric using mr imaging for preterm infants. AJNR Am J Neuroradiol.

[111] Kyriakopoulou V (2017). Normative biometry of the fetal brain using magnetic resonance imaging. Brain Struct Funct.

[112] Brouwer MJ (2017). Preterm brain injury on term-equivalent age mri in relation to perinatal factors and neurodevelopmental outcome at two years. PLoS One.

