# Supplementary material for: Cerebellar-dependent associative learning is impaired in very preterm born children and young adults
Source: Sci Rep. 2017 Dec 21;7:18028. doi: 10.1038/s41598-017-18316-8 (PMC5740078; doi:10.1038/s41598-017-18316-8)
Supplement: Supplementary file 1 — Supplementary Material [file 41598_2017_18316_MOESM1_ESM.pdf]

Supplementary Information

for the manuscript entitled:

Cerebellar-dependent associative learning is impaired in very preterm born children and young adults

Liliane Tran[#1], Britta M. Huening[#*1,2], Olaf Kaiser[1], Bernd Schweiger[3], Selma Sirin[3], Harald H. Quick[2,4], Ursula Felderhoff-Mueser[1], Dagmar Timmann[5]

[1] Department of Paediatrics I, Neonatology, Paediatric Intensive Care, Paediatric Neurology, University Hospital Essen, University of Duisburg-Essen, Essen, Germany

[2] Erwin L. Hahn Institute for Magnetic Resonance Imaging, University of Duisburg-Essen, Essen, Germany

[3] Institute of Diagnostic and Interventional Radiology and Neuroradiology, University Hospital Essen, University of Duisburg-Essen, Essen, Germany

[4] High Field and Hybrid MR Imaging, University Hospital Essen, University of Duisburg-Essen, Essen, Germany

[5] Department of Neurology, University Hospital Essen, University of Duisburg-Essen, Essen, Germany

[#]These authors contributed equally to this work

Supplementary Figure 1

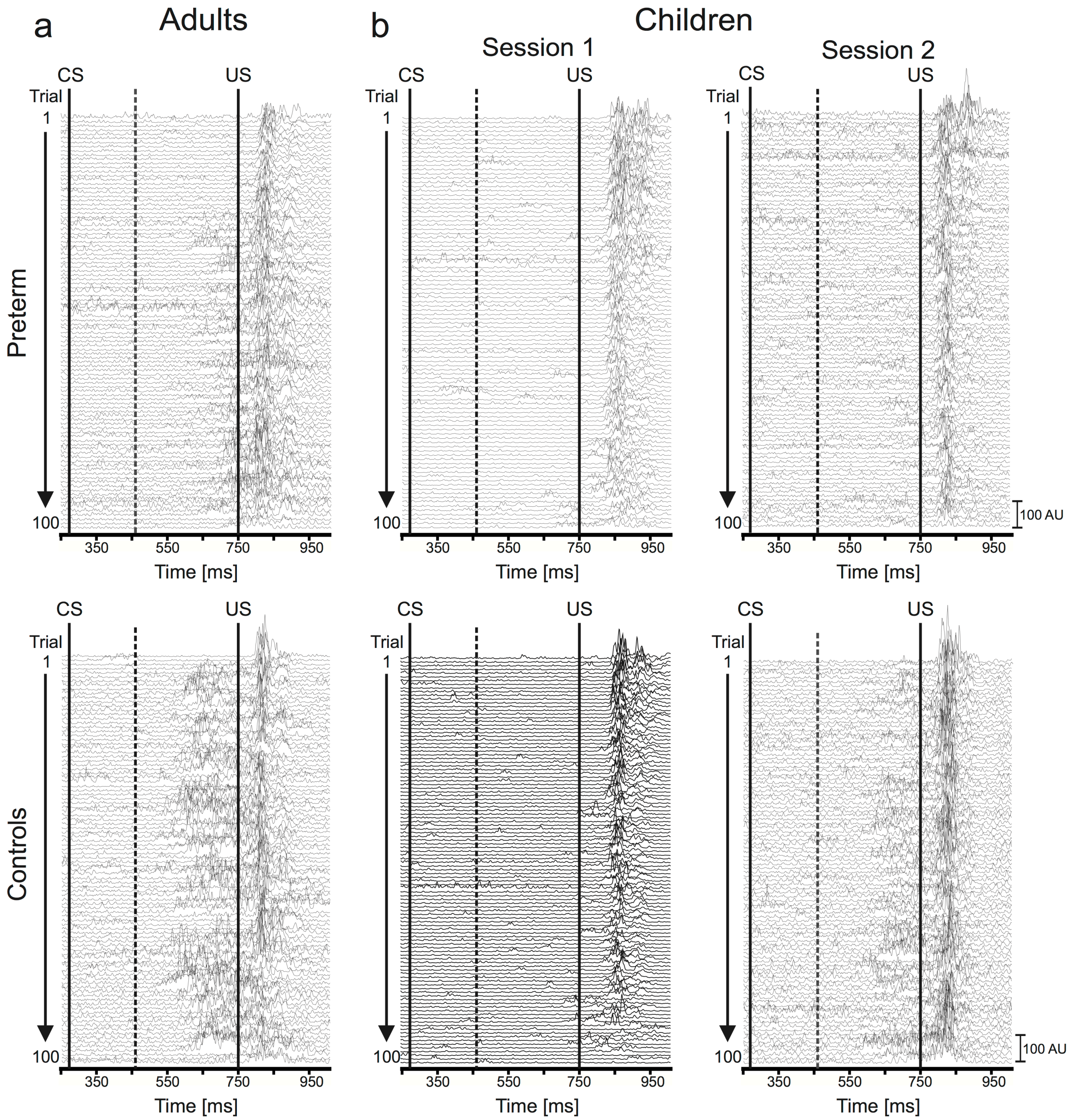

Eyeblink conditioning in characteristic preterm and control subjects.

Rectified and filtered EMG-data of the orbicularis oculi muscle are shown in (**a**) a preterm born adult (upper row) and control subject (lower row), and (**b**) a preterm child (upper row) and control child (lower row) in sessions 1 and 2. EMG-data are shown in the 100-paired CS-US acquisition trials with the first trial at the top and the last trial at the bottom. The first solid vertical line indicates the onset of the CS (the tone), the second solid vertical line indicates the onset of the US (the air puff). EMG responses occurring in the time interval of 150 ms after CS onset (indicated by the hatched line) and US onset were identified as CRs. See methods for further details. EMG: electromyography; CS = conditioned stimulus; US = unconditioned stimulus; CR = conditioned response.

Supplementary Figure 2

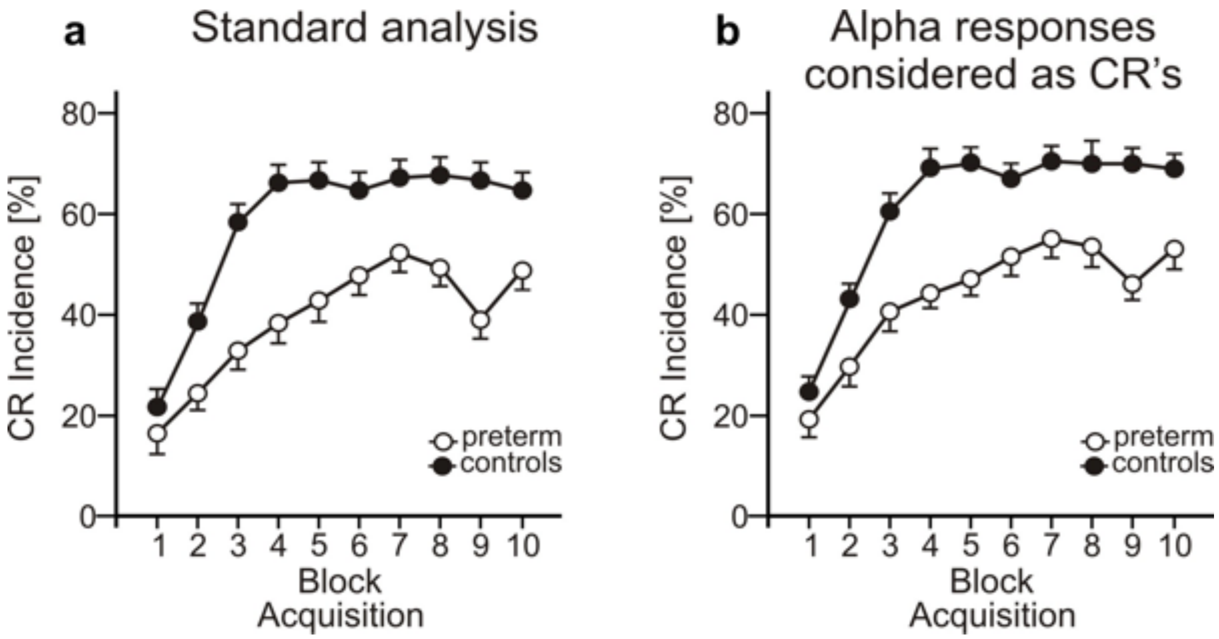

As outlined in Methods, responses occurring within the 150 ms interval after CS onset were defined as alpha responses (that is, reflexive responses to the tone) and not as CRs (Woodruff-Pak *et al*., 1996). Preterm born adults showed significantly more alpha responses than controls [5.8 (SD 3.99) in the preterm group vs. 3.7 (SD 1.83) in controls; $p = 0.04$, ANOVA]. To exclude that reduced CR acquisition in preterm born adults was caused by ill-timed CRs (i.e., CRs occurring so early that they were identified as alpha responses), analysis of CR acquisition was repeated with alpha responses being considered CRs. **Figure 2a** shows results of the standard analysis, that is with alpha responses being excluded (cf. Fig. 1 in the main manuscript). **Figure 2b** shows data of the additional analysis with alpha responses being considered CRs. Mean percentage conditioned response (CR) incidence and standard error (SE) are shown in preterm born adults (**o**) and control adults (•) across the ten acquisition blocks. Main findings were the same. Control adults acquired more conditioned responses than preterm born adults. Considering alpha responses as CR the group effect was significant [$F(1, 38) = 9.87$ $p = 0.003$] with preterm born adults acquiring significantly less conditioned responses than controls. The block by group interaction was not significant [$F(9, 342)= 1.34$, $p = 0.24$]. The block effect was significant [$F(9, 342) = 27.83$, $p < 0.001$].

Supplementary Figure 3

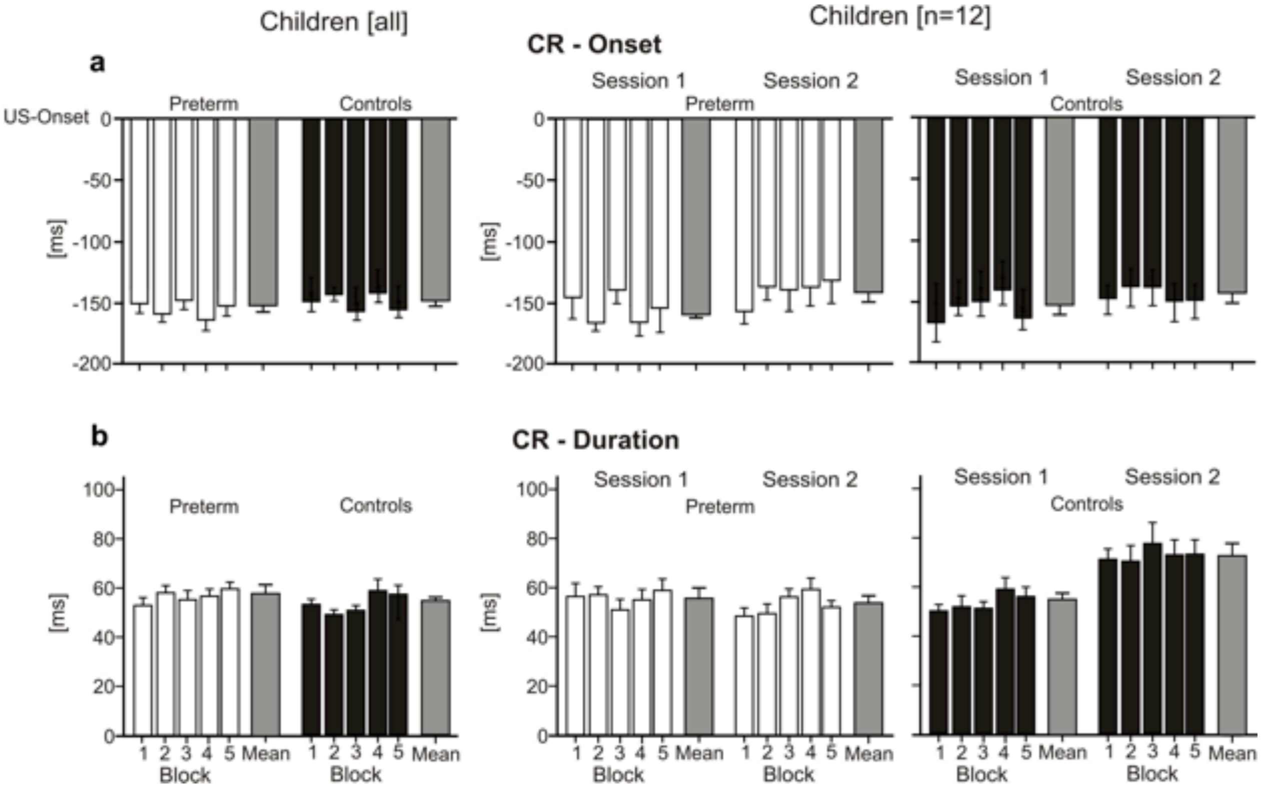

Timing and performance of conditioned eyeblink responses in children. Means and standard errors (SE) of (**a**) CR onset and (**b**) CR duration are shown in preterm children (open columns) and control children (filled columns). Data is shown in the groups of all children (first column; n = 32 per group) and in the subgroups tested twice (second column: session 1, third column: session 2; n = 12 per group). Note that (negative) values for CR onset refer to the time prior to the onset of the US (air puff), set as 0 ms. Each block corresponds to 20 CS-US paired acquisition trials. Mean values of all acquired CRs are shown in the grey column. In the subgroups tested twice, control children, but not preterm born children, increased CR duration across sessions [significant group and group by session interaction effects (see Supplementary Table 2 for details)].

Supplementary Table 1.

Summary of statistical findings comparing preterm born adults and matched controls. Significant differences for CR-incidence, spontaneous blink rate ($p < 0.05$), CR timing and performance ($p < 0.0125$; Bonferroni correction) and UR timing and performance ($p < 0.016$; Bonferroni correction) are highlighted in bold.

**Adult group**				
**ANOVA**	**Factors**		**F statistics**	***P***
**Conditioned responses (CR)**				
**CR incidence**				
Acquisition (Block 1-10)	between	Group	**F (1, 38) = 10.74**	**0.002**
	within	Block	**F (9, 342) = 30.18**	**< 0.001**
		Block*Group	**F (9, 342) = 2.19**	**0.02**
Extinction (last acquisition block, 3 extinction blocks)	between	Group	F (1, 37) = 2.45	0.13
	within	Block	**F (3, 111) = 48.17**	**< 0.001**
		Block*Group	F (3, 111) = 1.47	0.23
**CR timing and performance**				
Onset	between	Group	F (1, 34) = 0.0	0.99
	within	Block	F (4, 136) = 3.64	0.02
		Block*Group	F (4, 136) = 0.57	0.68
Peak time	between	Group	F (1, 34) = 1.73	0.20
	within	Block	**F (4, 136) = 6.14**	**0.001**
		Block*Group	F (4, 136) = 0.41	0.8
Area 50ms	between	Group	F (1, 34) = 0.27	0.6
	within	Block	**F (1, 43) = 10.65**	**0.004**
		Block*Group	F (1, 34) = 1.57	0.21
Duration	between	Group	F (1, 34) = 4.34	0.04
	within	Block	**F (4, 136) = 20.68**	**< 0.001**
		Block*Group	F (4, 136) = 0.80	0.47
**Spontaneous blink rate**				
	between	Group	F (1, 37) = 1.885	0.18
	within	Beginning vs end	F (1, 37) = 0.007	0.93
		Beginning vs end*Group	F (1, 37) = 0.007	0.93
**T-Test (unpaired)/Wilcoxon**	**Factors**		**T/Z-statistics**	***P***
**Unconditioned responses (UR)**				
Onset	between	Group	Z = -0.46, n = 20	0.65
Peak time	between	Group	T (38) = -1.43	0.16
Duration	between	Group	Z = -0.56, n = 20	0.57

Supplementary Table 2.

Summary of statistical findings comparing preterm children and matched controls.

Significant differences for CR-incidence, spontaneous blink rate (p < 0.05), CR timing and performance (p < 0.0125; Bonferroni correction) and UR timing and performance (p< 0.016; Bonferroni correction) are highlighted in bold.

**Children (all)**				
**ANOVA**	**Factors**		**F statistics**	***P***
**Conditioned responses (CR)**				
**CR incidence**				
Acquisition (Block 1-10)	between	Group	F (1, 62) = 2.19	0.14
	within	Block	F (9, 558) = 1.50	0.14
		Block*Group	F (9, 558) = 0.96	0.47
Extinction (last acquisition block, one extinction block)	between	Group	F (1, 62) = 0.003	0.95
	within	Block	**F (1, 62) = 14.17**	**< 0.001**
		Block*Group	F (1, 62) = 1.57	0.21
**CR timing and performance**				
Onset	between	Group	F (1, 57) = 0.46	0.50
	within	Block	F (4, 228) = 0.28	0.89
		Block*Group	F (4, 228) = 1.18	0.32
Peak time	between	Group	F (1, 57) = 0	0.98
	within	Block	F (4, 228) = 0.60	0.64
		Block*Group	F (4, 228) = 0.79	0.52
Area 50ms	between	Group	F (1, 56) = 0.05	0.83
	within	Block	F (1, 56) = 1.20	0.31
		Block*Group	F (1, 56) = 1.59	0.19
Duration	between	Group	F (1, 55) = 0.021	0.88
	within	Block	**F (4, 220) = 3.26**	**0.01**
		Block*Group	F (4, 220) = 0.56	0.69
**Spontaneous blink rate**				
	between	Group	F (1, 60) = 3.09	0.084
	within	Beginning vs end	F (1, 60) = 0.91	0.34
		Beginning vs end*Group	F (1, 60) = 0.14	0.70
**T-Test (unpaired)/Wilcoxon**	**Factors**		**T/Z-statistics**	***P***
**Unconditioned responses (UR)**				
Onset	between	Group	Z = -1.88, n = 32	0.06
Peak time	between	Group	**Z = -2.7, n = 32**	**0.007**
Duration	between	Group	Z = -1.85, n = 32	0.06
**Children session 1 vs session 2**				
**ANOVA**	**Factors**		**F statistics**	***P***
**Conditioned responses (CR)**				
**CR incidence**				
Acquisition (Block 1-10)	between	Group	**F (1, 22) = 6.57**	**0.02**
	within	Session	F (1, 22) = 4.07	0.06
		Block	**F (9, 198) = 3.20**	**0.001**
		Session*Group	F (1, 22) = 2.84	0.11
		Block*Group	F (9,198) = 0.74	0.67
		Session*Block	F (9, 198) = 0.88	0.55
		Session*Block*Group	F (9, 198) = 1.21	0.29
Extinction (last acquisition block, one extinction block)	between	Group	F (1, 22) = 3.92	0.06
	within	Session	F (1, 22) = 4.01	0.06
		Block	**F (1, 22) = 11.31**	**0.003**
		Session*Group	**F (1, 22) = 4.83**	**0.04**
		Block*Group	F (1, 22) = 0.01	0.94
		Session*Block	F (1, 22) = 0.21	0.65
		Session*Block*Group	F (1, 22) = 2.41	0.13

**CR timing and performance**				
Onset	between	Group	F (1, 20) = 0.06	0.81
	within	Session	F (1, 20) = 3.50	0.08
		Block	F(4, 80) = 0.34	0.85
		Session*Group	F (1, 20) = 0.03	0.86
		Block*Group	F (4, 80) = 0.358	0.85
		Session*Block	F (4, 80) = 0.74	0.57
		Session*Block*Group	F (4, 80) = 0.91	0.46
Peak time	between	Group	F (1, 20) = 0.30	0.59
	within	Session	**F (1, 20) = 7.68**	**0.01**
		Block	F (4, 80) = 0.68	0.57
		Session*Group	F (1, 20) = 0.32	0.57
		Block*Group	F (4, 80) = 0.36	0.84
		Session*Block	F (4, 80) = 0.65	0.58
		Session*Block*Group	F (4, 80) = 2.75	0.05
Area 50ms	between	Group	F (1, 18) = 0.31	0.58
	within	Session	**F (1, 18) = 14.10**	**0.001**
		Block	F (4, 72) = 0.70	0.54
		Session*Group	**F (1, 18) = 12.61**	**0.002**
		Block*Group	F (4, 72) = 0.29	0.81
		Session*Block	F (4, 72) = 0.54	0.71
		Session*Block*Group	F (4, 72) = 0.82	0.52
Duration	between	Group	**F (1, 20) = 10.84**	**0.004**
	within	Session	F (1,20) = 6.57	0.02
		Block	F (4, 80) = 1.51	0.21
		Session*Group	**F (1, 20) = 9.91**	**0.005**
		Block*Group	F (4, 80) = 0.07	0.99
		Session*Block	F (4, 80) = 0.75	0.56
		Session*Block*Group	F (4, 80) = 1.24	0.3
**Spontaneous blink rate**				
	between	Group	**F (1, 22) = 4.32**	**0.049**
	within	Session	**F (1, 22) = 23.23**	**< 0.001**
		Beginning/end	F (1, 22) = 0.13	0.71
		Session*Group	F (1, 22) = 2.35	0.13
		Beginning/end*Group	F (1, 22) = 0.61	0.44
		Session*Beginning/end	F (1, 22) = 0.36	0.55
		Session*Beginning/end*Group	F (1, 22) = 1.14	0.29
**Unconditioned responses (UR)**				
Onset	between	Group	F (1, 22) = 6.0	0.02
	within	Session	F (1, 22) = 0.16	0.69
		Group*Session	F (1, 22) = 1.04	0.32
Peak time	between	Group	F (1, 22) = 0.34	0.57
	within	Session	F (1, 22) = 0.03	0.85
		Group*Session	F (1, 22) = 2.61	0.12
Duration	between	Group	F (1, 21)= 3.7	0.07
	within	Session	F (1, 21)= 1.15	0.3
		Group*Session	F (1, 21)= 0.72	0.41
